# Histomorphological changes in human septal cartilage stored in different preservation solutions

**DOI:** 10.55730/1300-0144.6366

**Published:** 2026-07-21

**Authors:** Özlem AKKOCA, Akif Sinan BİLGEN, Harun ÇOBAN, Fatma ASLAN YAY, Zeynep KAPTAN, Funda BALKILIÇ AKKAYA, Rahmi KILIÇ, Sami Engin MUZ

**Affiliations:** 1 Department of Otorhinolaryngology, University of Health Sciences, Ankara Training and Research Hospital, Ankara, Turkiye; 2 Department of Pathology, University of Health Sciences, Sakarya Training and Research Hospital, Sakarya, Turkiye

**Keywords:** Cartilage, histomorphology, tissue preservation, chondrocytes, ethanol, povidone-iodine

## Abstract

**Background/aim:**

To assess the histopathological effects of three preservation solutions (70% ethanol, povidone-iodine, and ciprofloxacin) on human septal cartilage obtained during septoplasty and to compare their preservation characteristics for potential future graft applications. Optimal preservation of septal cartilage is clinically important because well-preserved grafts may reduce the need for secondary cartilage harvesting and the associated donor-site morbidity while facilitating reconstructive procedures during revision surgery.

**Materials and methods:**

A total of 90 septal cartilage specimens obtained from 30 patients undergoing septoplasty were allocated to three preservation groups and stored in the designated preservation solutions at +4 °C. Samples were examined histologically on days 0, 15, and 30. The evaluated parameters included chondrocyte count/cellularity, the percentage of empty lacunae, chondrocyte morphology, erosion score, inflammation, and necrosis. Evaluations were blinded.

**Results:**

Progressive time-dependent histomorphological deterioration was observed in all preservation groups. From baseline to day 30, the ciprofloxacin group showed a greater reduction in chondrocyte count and a greater increase in the percentage of empty lacunae than the povidone-iodine group (adjusted p = 0.043 and p = 0.029, respectively). Although ethanol showed relatively favorable morphological findings at day 15, substantial deterioration was observed by day 30. Chondrocyte morphology and erosion scores also deteriorated over time. Inflammation and necrosis remained minimal across all groups.

**Conclusion:**

All tested preservation solutions were associated with progressive, time-dependent histomorphological deterioration of septal cartilage. No single preservation solution demonstrated consistently superior preservation across all evaluated histopathological parameters. Further biomechanical and biochemical studies are required to establish optimal cartilage preservation protocols.

## 1. Introduction

Cartilage grafts are widely used in reconstructive procedures due to their structural stability and favorable biomechanical properties. Preservation of harvested cartilage tissue is clinically important, particularly for secondary or revision surgeries, in which the availability of autologous grafts may reduce donor-site morbidity and improve surgical outcomes [1].

Among various sources of autologous cartilage, the nasal septum represents a commonly used and easily accessible donor site in otolaryngological surgery. However, there is currently no standardized protocol for the optimal preservation of septal cartilage intended for future graft use [2].

An ideal storage medium should maintain both extracellular matrix integrity and chondrocyte viability while minimizing microbial contamination and preventing structural degradation. Various chemical agents have been used for this purpose in clinical practice. Ethanol is widely used for its antimicrobial properties, although its dehydrating effects on the cartilage matrix remain a concern [1,2]. Similarly, povidone-iodine and ciprofloxacin are commonly used as antiseptic and antibiotic agents, respectively; however, both have been associated with potential cytotoxic effects on chondrocytes and structural deterioration of cartilage tissue [3,4].

Despite their frequent clinical use, comparative histomorphological data regarding the long-term effects of these agents on human septal cartilage remain limited. Therefore, this study aimed to evaluate and compare the histopathological changes in human nasal septal cartilage stored in ethanol, povidone-iodine, and ciprofloxacin solutions, with particular emphasis on histomorphological changes and structural integrity over time.

## 2. Materials and methods

### 2.1. Study design and sample selection

This prospective observational histopathological study used nasal septal cartilage specimens obtained as surgical waste during septoplasty procedures. Ethical approval was obtained from the Ankara Training and Research Hospital Ethics Committee (approval no: E-25-528).

Between June 2025 and November 2025, septal cartilage specimens obtained from 30 patients undergoing septoplasty were divided into three equal specimens per patient, yielding a total of 90 cartilage specimens for analysis. Septal deviation was evaluated preoperatively with anterior rhinoscopy, flexible fiberoptic nasopharyngoscopy, and paranasal sinus computed tomography.

A total of 42 patients were assessed for eligibility. Based on the predefined clinical eligibility criteria, 12 patients were excluded due to bleeding disorders (n = 1), autoimmune thyroiditis (n = 2), diabetes mellitus (n = 3), previous nasal surgery (n = 3), or use of acetylsalicylic acid or nonsteroidal antiinflammatory drugs (n = 3). The remaining 30 patients were enrolled in the study. These criteria were applied during participant selection to obtain a more clinically homogeneous tissue sample; however, the direct effects of these systemic conditions or medications on ex vivo septal cartilage behavior were not evaluated in the present study.

### 2.2. Sample preparation and group allocation

All patients underwent surgery under general anesthesia, following a standard protocol. Anesthesia was induced with midazolam and remifentanil and was maintained with sevoflurane and remifentanil. The septal mucosa was infiltrated with 1:100,000 epinephrine-lidocaine for local hemostasis.

After correction of the septal pathology, nine equal cartilage strips measuring approximately 3–4 mm each were prepared from each resected septal cartilage specimen. The specimens were then randomly assigned to one of three preservation solution groups using a simple randomization method.

- Ethanol solution (70% ethanol): Samples were immersed for the designated storage periods and rinsed with sterile phosphate-buffered saline (PBS) prior to analysis.
- Ciprofloxacin solution: Sterile PBS supplemented with 0.2 mg/mL ciprofloxacin prepared from an intravenous stock solution was used for specimen storage.
- Povidone-iodine solution (10% povidone-iodine): Samples were gently rinsed with PBS before histopathological evaluation.

All cartilage specimens were stored at +4 °C throughout the 30-day study period, regardless of the preservation solution used.

The three solutions were selected based on their antimicrobial properties, ready availability, and low cost, rather than their pharmacological class. The ciprofloxacin concentration was set at 0.2 mg/mL based on previously reported experimental data [5].

Within each preservation solution group, specimens were evaluated at three predefined time points to assess time-dependent changes.

- Day 0 (baseline)
- Day 15
- Day 30

At the end of each designated storage period, specimens were fixed in 10% neutral buffered formalin for histopathological evaluation.

### 2.3. Histopathological examination

After routine tissue processing, paraffin blocks were prepared, and sections measuring 4–5 μm in thickness were cut. Sections were stained with hematoxylin and eosin (H&E). All histopathological evaluations were performed by a pathologist blinded to the group assignments. Examinations were performed using a light microscope (Olympus BX51; Olympus Corporation, Tokyo, Japan). In each specimen, three fields were evaluated at 20× magnification and averaged. For detailed evaluation of cellular morphology, 40× magnification was used.

### 2.4. Evaluated histopathological parameters

Histopathological evaluation was performed using six parameters to assess cartilage cellularity and histomorphological integrity.

#### 2.4.1. Chondrocyte count/cellularity

At 20× magnification, chondrocytes with clearly visible nuclei within the lacunae were counted in the evaluated histological fields. Lacunae without a visible nucleus were excluded from the chondrocyte count. The number of nucleated chondrocytes was recorded as the chondrocyte count/cellularity.

#### 2.4.2. Empty lacunae percentage

The percentage of lacunae without a visible nucleus was calculated relative to the total number of evaluated lacunae.

#### 2.4.3. Chondrocyte morphology score

Degenerative cellular changes were graded as follows: 0, normal chondrocyte morphology; 1, mild nuclear pyknosis or cellular shrinkage; 2, marked nuclear pyknosis with cytoplasmic contraction; and 3, extensive cell death and severe morphological distortion.

#### 2.4.4. Erosion score

Cartilage surface irregularities and erosive changes were scored as follows: 0, smooth and intact surface; 1, mild surface irregularity; 2, distinct fissures or focal erosions; and 3, extensive surface disruption and tissue loss.

#### 2.4.5. Inflammation

Perichondral or intratissue inflammation was graded as follows: 0, no inflammation; 1, mild inflammatory cell presence; 2, moderate inflammation; and 3, severe inflammatory infiltration.

#### 2.4.6. Necrosis

Based on the findings of cellular loss and coagulative necrosis, necrosis was scored as follows: 0, no necrosis; 1, focal necrotic areas; 2, moderately extensive necrosis; and 3, widespread severe necrosis.

### 2.5. Statistical analysis

Descriptive statistics for continuous variables are presented as mean ± standard deviation, median, minimum, and maximum values. Categorical variables are shown as counts and percentages. Data normality was assessed using the Shapiro–Wilk test. Differences among cartilage samples within each preservation solution at days 0, 15, and 30 were analyzed using Friedman’s repeated-measures ANOVA. Post hoc comparisons were performed using Friedman’s multiple comparisons test to determine which time points contributed to the observed differences, and adjusted p values were reported. Changes from day 0 to days 15 and 30 across the different preservation solutions were also analyzed using Friedman’s repeated-measures ANOVA, followed by post hoc multiple comparisons with adjustment for multiple testing. Changes were defined as follows: Δ1 = Day 15 − Day 0, Δ2 = Day 30 – Day 0, and Δ3 = Day 30 – Day 15. A priori sample size calculation or power analysis was not performed for this study because it was designed as a preliminary observational investigation. All analyses were conducted using IBM SPSS Statistics for Windows, version 20.0 (IBM Corp., Armonk, NY, USA), and p < 0.05 were considered statistically significant.

## 3. Results

### 3.1. Ethanol group

**Figure 1:**
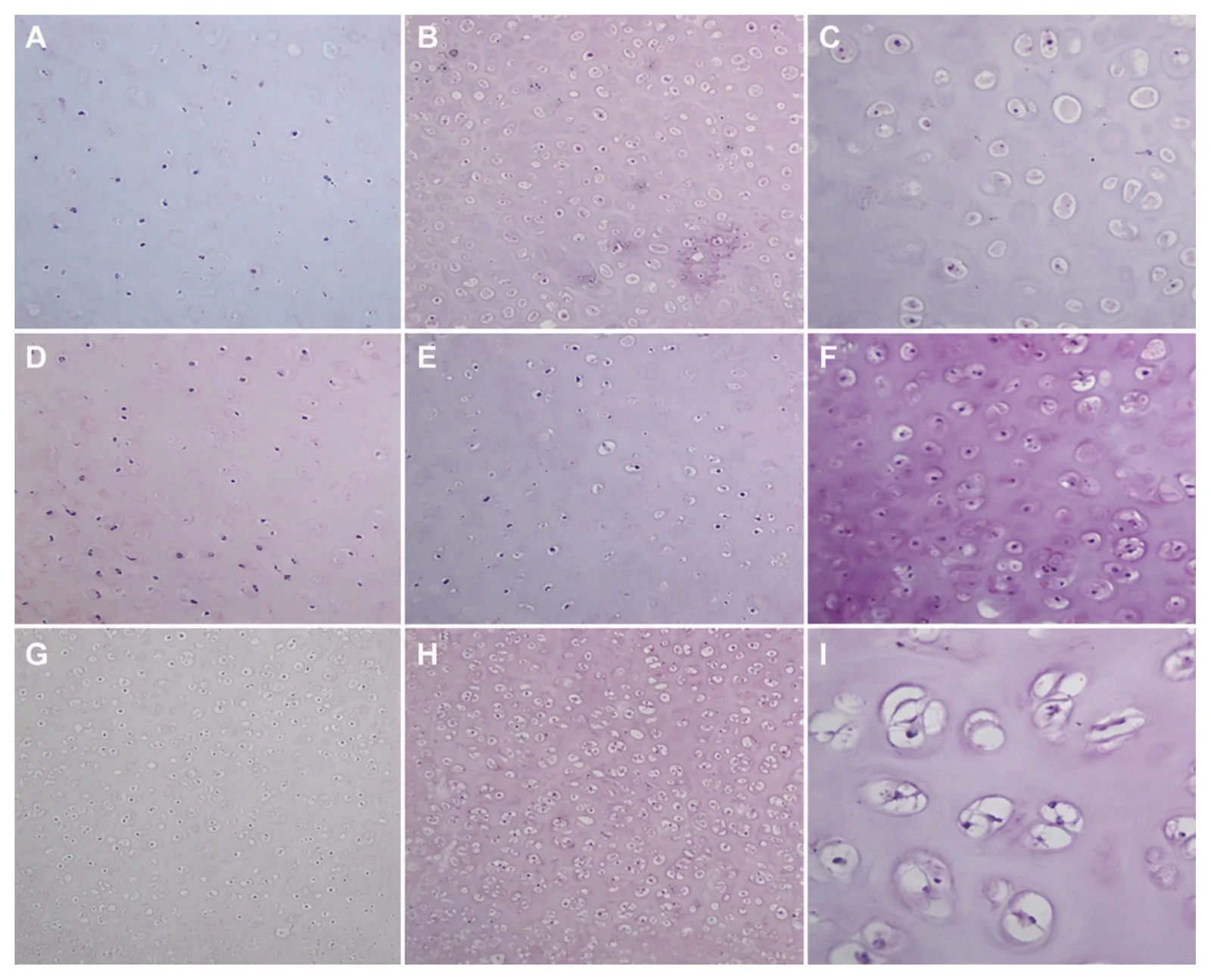
Representative hematoxylin and eosin-stained sections of septal cartilage specimens stored in different preservation solutions at days 0, 15, and 30. (A–C) Ethanol, (D–F) povidone-iodine, and (G–I) ciprofloxacin; each column represents days 0, 15, and 30, respectively. At day 0, all groups demonstrated preserved chondrocytes within well-defined lacunae. At days 15 and 30, progressive chondrocyte loss and an increased percentage of empty lacunae were observed, most prominently in the ciprofloxacin group, whereas degenerative changes were less pronounced in the ethanol and povidone-iodine groups (H&E, ×200).

Compared to baseline (day 0), chondrocyte counts decreased on day 15 (p < 0.05) and day 30 (p < 0.001). Chondrocyte counts were lower on day 30 than on day 15 (p < 0.01). The percentage of empty lacunae increased on day 15 (p < 0.05) and day 30 (p < 0.001) compared with day 0 (Figure 1A–1C). Chondrocyte morphology scores were higher on day 30 than on day 0 (p < 0.001) and day 15 (p < 0.01), whereas no significant difference was observed between day 0 and day 15. Erosion scores increased on day 30 compared with day 0 (p < 0.05), whereas no significant differences were observed at the other time points (Table 1).

### 3.2. Povidone-iodine group

Chondrocyte counts significantly decreased on day 15 (p < 0.01) and day 30 (p < 0.001) compared with baseline. Chondrocyte counts were significantly lower on day 30 than on day 15 (p < 0.01). The percentage of empty lacunae was significantly higher on day 15 (p < 0.05) and day 30 (p < 0.001) than at baseline and showed a further increase on day 30 compared with day 15 (p < 0.001) (Figure 1D–1F). Chondrocyte morphology scores were significantly higher on day 30 than on day 0 (p < 0.001), whereas no significant differences were observed between the other time points. Erosion scores were significantly higher on day 30 than on day 0 (p < 0.01), whereas no significant differences were detected at the other time points (Table 2).

### 3.3. Ciprofloxacin group

Chondrocyte counts significantly decreased on day 15 (p < 0.01) and day 30 (p < 0.001) compared with baseline. Chondrocyte counts were also significantly lower on day 30 than on day 15 (p < 0.01). The percentage of empty lacunae significantly increased on day 15 (p < 0.01) and day 30 (p < 0.001) compared with day 0, with a further increase observed on day 30 compared with day 15 (p < 0.01) ( Figure 1G–1I). Chondrocyte morphology scores were significantly higher on day 30 than on day 0 (p < 0.001), whereas no significant differences were found among the other time points. Erosion scores were significantly higher on day 30 than on day 0 (p < 0.01), with no significant differences among the other time points (Table 3).

The change in chondrocyte count from baseline to day 30 (Δ2) differed significantly among the preservation solutions (p = 0.048). Adjusted pairwise comparisons showed a greater decrease in chondrocyte count in the ciprofloxacin group than in the povidone-iodine group (adjusted p = 0.043). Similarly, the change in the percentage of empty lacunae from baseline to day 30 (Δ2) differed significantly among the solutions (p = 0.032), with a greater increase in the ciprofloxacin group than in the povidone-iodine group (adjusted p = 0.029). Although the overall between-solution comparison of the change in chondrocyte morphology score from day 15 to day 30 (Δ3) was statistically significant (p = 0.035), none of the pairwise comparisons remained statistically significant after adjustment for multiple comparisons. No significant between-solution differences were observed for the remaining change values, including erosion scores (all p > 0.05) (Table 4).

### 3.4. Inflammation and necrosis

No inflammatory cell infiltration was seen in the ethanol and povidone-iodine groups at any time point. In the ciprofloxacin group, inflammatory cell scores did not differ significantly across time points (p > 0.05). Necrosis was present in only one specimen on days 15 and 30 in the ethanol group. No necrosis was found in the povidone-iodine group. Necrosis scores did not vary significantly over time in the ciprofloxacin group (p > 0.05).

**Figure 2:**
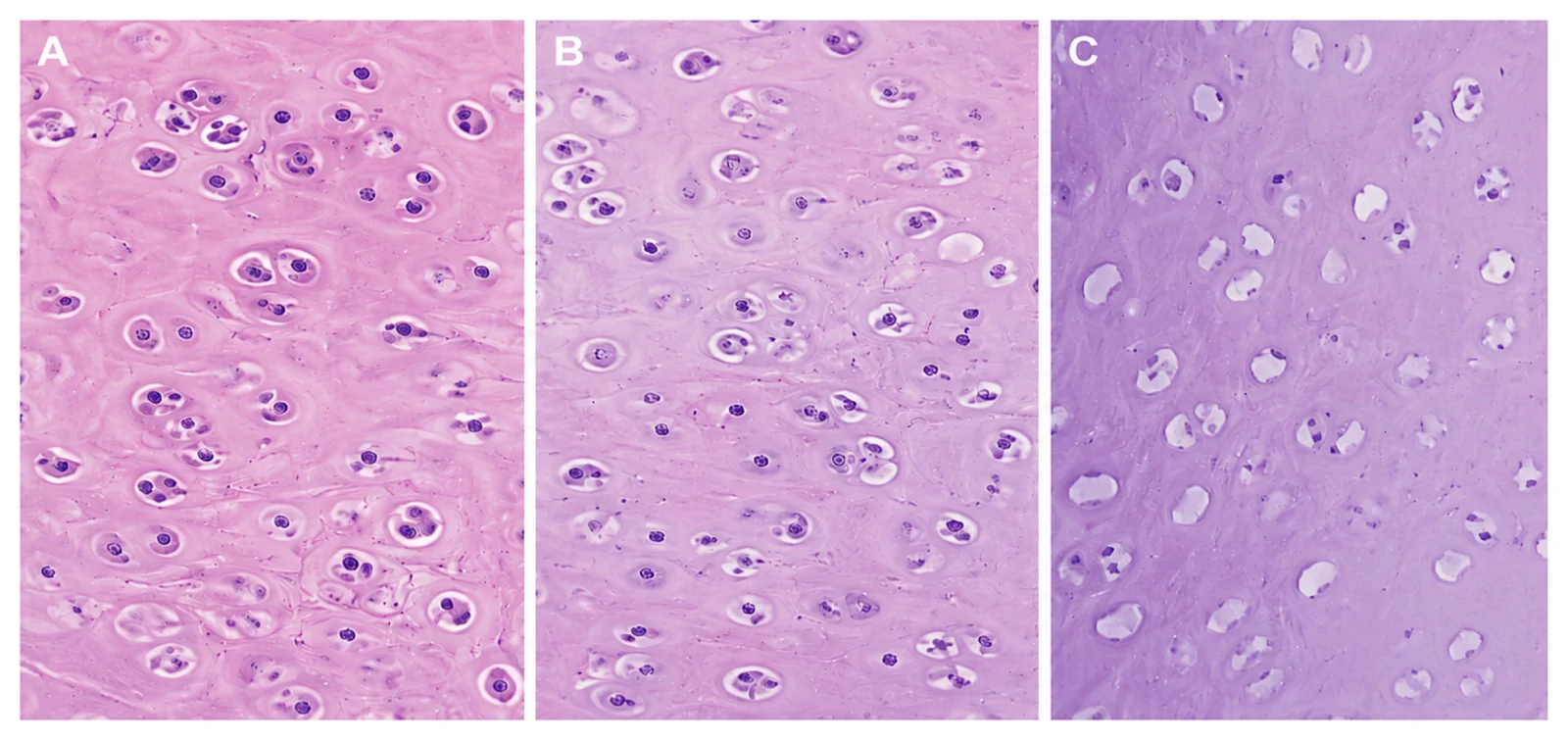
High-magnification hematoxylin and eosin-stained sections demonstrating degenerative morphological changes in septal cartilage specimens after 30 days of storage in different preservation solutions. (A) Ethanol, (B) povidone-iodine, and (C) ciprofloxacin. Chondrocytes exhibited degenerative morphological changes, including nuclear pyknosis, cellular shrinkage, and reduced cytoplasmic volume. Empty lacunae were also observed, reflecting chondrocyte loss. These morphological changes were most pronounced in the ciprofloxacin group (H&E, ×400).

Chondrocytes exhibited degenerative morphological changes, including nuclear pyknosis, cellular shrinkage, and reduced cytoplasmic volume. Empty lacunae were also frequently observed, reflecting chondrocyte loss. These morphological changes were more prominent in samples stored for longer durations, particularly in the ciprofloxacin group (H&E stain; ×400) (Figure 2).

**Figure 3:**
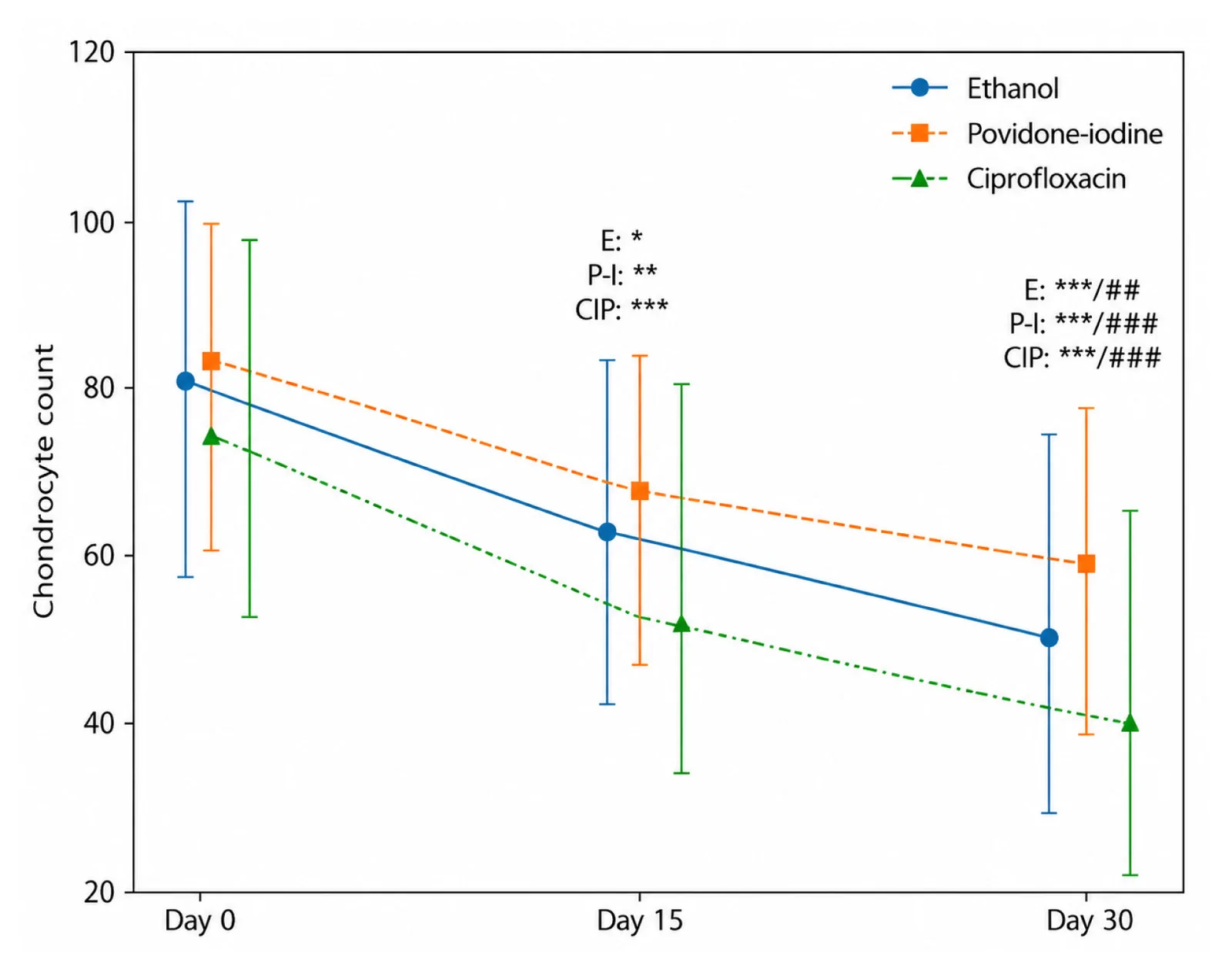
Changes in chondrocyte counts over time (days 0, 15, and 30). Error bars represent the standard deviation. *p < 0.05, **p < 0.01, ***p < 0.001 versus day 0; ##p < 0.01 and ###p < 0.001 versus day 15.

**Figure 4:**
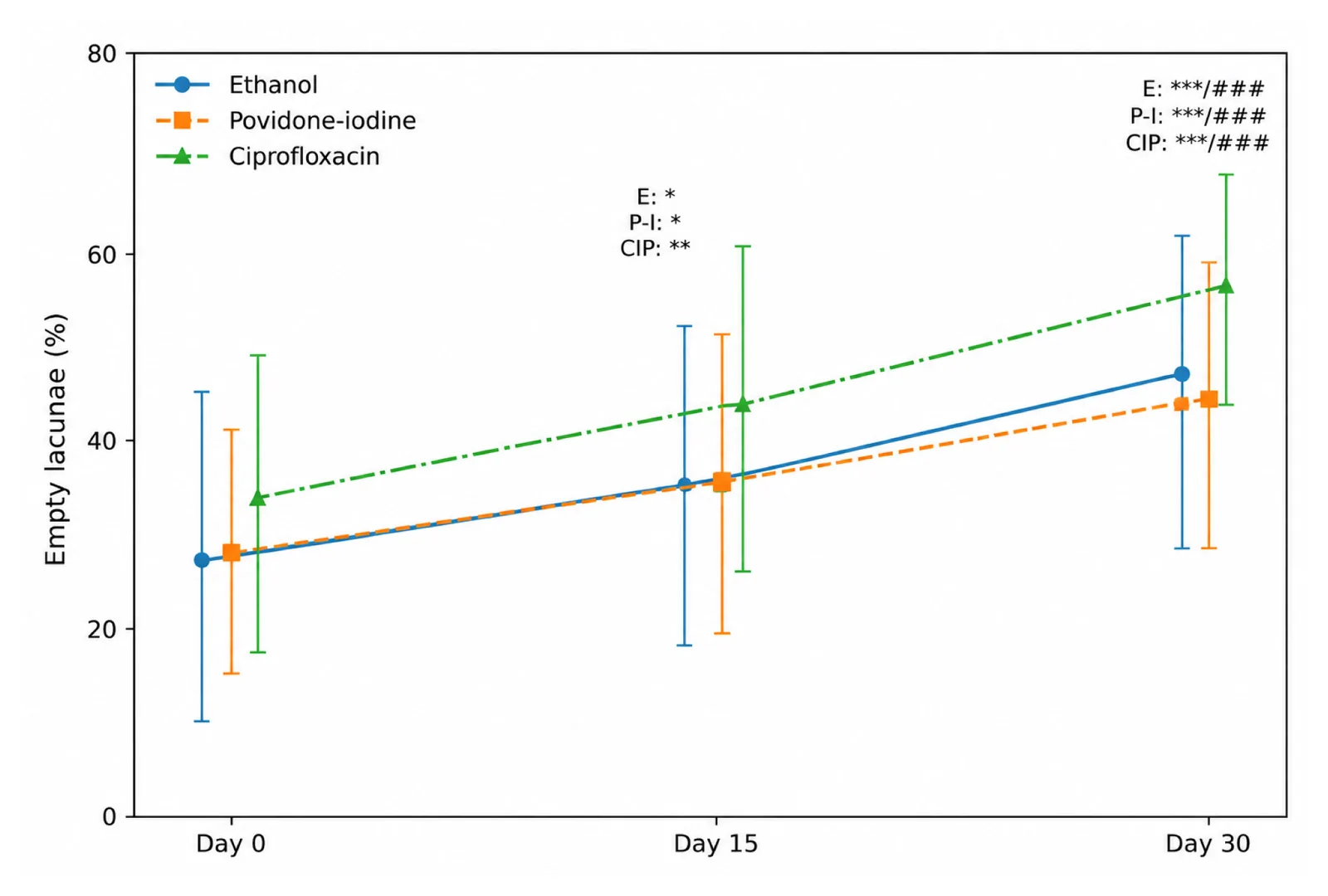
Percentage of empty lacunae over time (days 0, 15, and 30). Error bars represent the standard deviation. *p < 0.05, **p < 0.01, ***p < 0.001 versus day 0; ##p < 0.01 and ###p < 0.001 versus day 15.

Overall, all three solutions showed a time-dependent decrease in chondrocyte counts (Figure 3) and an increase in the percentage of empty lacunae (Figure 4).

## 4. Discussion

Cartilage graft preservation is a critical issue in reconstructive surgery, as maintaining cellular viability is essential for long-term graft function and structural integrity. In clinical practice, portions of nasal septal cartilage are frequently harvested during septorhinoplasty procedures while preserving the L-strut to maintain nasal support. However, if revision surgery becomes necessary, the need for additional autologous cartilage grafts may present a significant clinical challenge.

The most commonly used donor sites for autologous cartilage grafts include the nasal septum, auricular cartilage, and costal cartilage. Costal cartilage is the largest available source of cartilage in the body and can be easily shaped and harvested in large quantities. However, compared with other donor sites, rib cartilage harvesting is more invasive, results in a visible scar, and may cause serious complications, such as pneumothorax. Additionally, calcification of costal cartilage, particularly in elderly patients, constitutes another important limitation [4].

Therefore, saving excised septal cartilage for potential future revision procedures offers significant intraoperative convenience. In this context, optimal storage conditions are critical. An ideal storage method should preserve cartilage structure without causing inflammation, be readily accessible, and be cost-effective. In our study, because of their availability and low cost, cartilage specimens were stored in ethanol, povidone-iodine, and ciprofloxacin solutions, and histopathological examination was performed on days 0, 15, and 30.

A 2016 study evaluating septorhinoplasty patients reported revision surgery rates of 3.1% in primary cases and 11.0% in secondary cases. The same study demonstrated that revision rates increased with greater graft harvesting and reconstructive complexity. Among different graft types, septal cartilage had the lowest revision rate (6.3%), followed by bone grafts (11.0%), conchal grafts (11.4%), and rib grafts (21.5%) [6]. These findings emphasize the clinical importance of septal cartilage as a graft material in septorhinoplasty. However, there is no clear consensus in the literature regarding optimal in vitro preservation methods for septal cartilage. To our knowledge, this is the first study to directly compare ethanol, povidone-iodine, and ciprofloxacin as cartilage storage solutions.

In the ciprofloxacin group, a significant decrease in chondrocyte numbers and an increase in the number of empty lacunae were observed on day 15, with further deterioration by day 30. Similarly, chondrocyte morphology and erosion scores were significantly worse on day 30 compared with baseline. These findings indicate progressive cellular loss and structural deterioration during storage in ciprofloxacin solution.

Fluoroquinolones, including ciprofloxacin, have long been linked to chondrotoxic effects. Experimental studies have demonstrated decreased chondrocyte viability, suppression of proteoglycan synthesis, mitochondrial damage, and increased apoptosis following fluoroquinolone exposure [7–9]. Previous experimental literature has identified 0.2 mg/mL as a relevant threshold in the evaluation of fluoroquinolone-associated chondrotoxicity, with chondrotoxic effects generally not reported at concentrations below this level in animal cartilage models [5]. The ciprofloxacin concentration of 0.2 mg/mL was therefore selected because its histomorphological effects on human septal cartilage remain unclear. Nevertheless, fluoroquinolone-associated cartilage damage has been linked to oxidative stress, lipid peroxidation, and activation of inflammatory mediators such as IL-1β, TNF-α, and IL-6 through reactive oxygen species pathways [10,11]. Although most previous studies have focused on articular cartilage, the fundamental cellular composition of hyaline cartilage suggests that similar mechanisms may occur in septal cartilage. Therefore, the progressive histopathological deterioration observed in the ciprofloxacin group is consistent with the known inhibitory effects of fluoroquinolones on chondrocyte metabolism and cartilage integrity.

Pimenta et al. (2023) evaluated the effects of povidone-iodine lavage on rabbit knee cartilage and observed toxic effects after 60 days [12]. Similarly, in our study, the povidone-iodine group showed a time-dependent decrease in chondrocyte numbers and an increase in the percentage of empty lacunae. By day 30, there was clear deterioration in chondrocyte morphology, and erosion scores were evident.

Although povidone-iodine is widely used as a broad-spectrum antiseptic in surgical practice, experimental studies have demonstrated its cytotoxic effects on cartilage tissue. Even diluted solutions have been shown to reduce chondrocyte viability and disrupt extracellular matrix organization in articular cartilage [13–15]. The progressive cellular loss and morphological impairment observed in our study support these findings and suggest that prolonged exposure of septal cartilage to povidone-iodine may adversely affect cartilage cellularity and histomorphological integrity.

A 2025 study investigating cartilage preservation at +4 °C and −18 °C in various solutions, including formalin, alcohol, saline, gentamicin, and cefazolin sodium, over a 6-month period concluded that alcohol was associated with less necrosis and loss of metachromasia, suggesting superior preservation properties. The same study reported that storage temperature did not significantly influence cartilage preservation [4]. The day 0, day 15, and day 30 time points were selected to evaluate early and progressive short-term histomorphological changes under the tested storage conditions. The 30-day observation period was intentionally limited to short-term preservation and was not intended to model long-term tissue banking. Although previous studies have evaluated cartilage preservation for up to 6 months [4], the present study focused on identifying early tissue changes and differences among the tested solutions. Therefore, the findings of the present study should be interpreted as reflecting short-term preservation and should not be directly extrapolated to long-term cartilage storage.

In our study, the ethanol group demonstrated cartilage morphology scores comparable to baseline at day 15, while a significant increase was observed by day 30. These findings suggest that cellular destruction and structural deterioration may begin after 15 days of exposure and become statistically significant by day 30. However, the day 30 chondrocyte morphology score was numerically higher in the ethanol group than in the povidone-iodine and ciprofloxacin groups. Moreover, although the overall between-solution comparison of the change in morphology score from day 15 to day 30 was significant, no pairwise comparison remained significant after adjustment for multiple comparisons. Therefore, the relatively favorable findings observed for ethanol at day 15 should not be interpreted as evidence of consistently superior preservation across all histopathological parameters or storage durations.

When all three groups were evaluated together, a time-dependent decrease in chondrocyte counts and an increase in the percentage of empty lacunae were observed, indicating progressive cellular degeneration regardless of the storage solution. Notably, no significant inflammatory response was detected in any group, and necrosis was minimal or absent. Although marked degeneration and loss of chondrocytes were observed in all groups, the minimal inflammation and necrosis may be explained by the in vitro nature of the study, which excluded systemic immune responses and limited the presence of inflammatory mediators. These findings suggest that the observed histopathological changes are primarily attributable to the direct chemical effects of the preservation solutions rather than secondary inflammatory processes. The gradual increase in the percentage of empty lacunae with prolonged storage is consistent with progressive chondrocyte loss; however, the underlying mechanism of these morphological changes cannot be determined based on H&E evaluation alone. The histomorphological endpoints evaluated in this study may not directly correlate with clinical graft performance. Cartilage grafts provide substantial structural support through their extracellular matrix, and grafts with reduced or absent cellular viability may still retain clinical utility. Therefore, the observed reductions in chondrocyte counts and changes in cellular morphology should not be interpreted as direct evidence of impaired graft function or clinical failure. As biomechanical properties and in vivo graft outcomes were not assessed, the clinical significance of these histomorphological findings remains uncertain.

Baseline values were not completely uniform across the preservation groups. The nonzero morphology and erosion scores at day 0, as well as the modest differences in baseline chondrocyte counts, may reflect inherent histological heterogeneity within septal cartilage specimens, variation in sectioning and microscopic field selection, or tissue-processing effects. Although specimens from each patient were randomly allocated among the three preservation conditions, randomization cannot fully eliminate local differences between cartilage fragments. To reduce the influence of these baseline imbalances, between-solution comparisons were therefore based on change-from-baseline values rather than absolute follow-up values.

The study’s limitations include its in vitro design, short observation period, evaluation of only three storage solutions, a focus on histopathology without biochemical or biomechanical analyses, and a limited sample size. Another important limitation is the absence of an untreated or neutral saline/PBS control group. Therefore, the contribution of time-dependent ex vivo degeneration cannot be fully distinguished from changes associated with the tested preservation conditions. Additionally, the semiquantitative histopathological scores were assessed by a single blinded pathologist, and intraobserver and interobserver reliability were not evaluated, which may limit the reproducibility of the subjective scoring parameters. These limitations may restrict the generalizability and clinical applicability of our findings.

Future studies should address these limitations by conducting in vivo research to evaluate the biological behavior and integration of preserved cartilage within living tissues. Additionally, investigation of a broader range of storage solutions, including newer preservative agents or combinations, could identify more effective preservation techniques. Long-term studies incorporating biochemical assays and biomechanical testing are also recommended to assess the functional integrity of preserved cartilage. Such research would help establish evidence-based protocols and improve clinical outcomes in cartilage grafting.

## 5. Conclusion

All tested preservation solutions were associated with progressive, time-dependent histomorphological deterioration of septal cartilage. At day 30, ciprofloxacin showed a greater reduction in chondrocyte counts and a greater increase in the percentage of empty lacunae compared with povidone-iodine. However, no single preservation solution demonstrated consistently superior preservation across all evaluated histopathological parameters. Further biomechanical and biochemical studies are required to establish optimal cartilage preservation protocols.

## Figures and Tables

**Table 1:** Comparison of septal cartilage specimens stored in ethanol at days 0, 15, and 30 according to histopathological parameters.

	Day 0	Day 15	Day 30	
Ethanol	Mean ± SD Median (min–max)	Mean ± SD Median (min–max)	Mean ± SD Median (min–max)	p
Chondrocyte count/cellularity	80.60 ± 25.32 80 (35–123)	64.47 ± 18.43 65 (33–96)	53.70 ± 19.79 44 (28–98)	**<0.001** b
Empty lacunae %	27.17 ± 18.80 21 (3–67)	34.90 ± 18.16 33 (5–71)	48.47 ± 19.65 55 (6–76)	**<0.001** b
Chondrocyte morphology score	0.90 ± 0.66 1 (0–2)	1.27 ± 0.74 1 (0–3)	2.00 ± 0.58 2 (0–3)	**<0.001** b
Erosion score	0.33 ± 0.47 0 (0–1)	0.63 ± 0.66 1 (0–2)	0.77 ± 0.67 1 (0–2)	**<0.001** b
Inflammation	0 (0–0)	0 (0–0)	0 (0–0)	**-**
Necrosis	0 (0–0)	0 (0–1)	0 (0–1)	0.368 b

b Overall p-values obtained from Friedman’s repeated-measures analysis of variance by ranks.

Bold values indicate statistical significance (p < 0.05).

**Table 2:** Comparison of septal cartilage specimens stored in povidone-iodine at days 0, 15, and 30 according to histopathological parameters.

	Day 0	Day 15	Day 30	
Povidone-iodine	Mean ± SD Median (min–max)	Mean ± SD Median (min–max)	Mean ± SD Median (min–max)	p
Chondrocyte count/cellularity	82.47 ± 21.09 81 (45–124)	68.80 ± 18.67 67 (42–104)	61.40 ± 18.70 55 (38–96)	**<0.001** b
Empty lacunae %	27.67 ± 13.98 31 (3–55)	36.03 ± 16.70 41 (8–62)	45.47 ± 17.46 49 (15–73)	**<0.001** b
Chondrocyte morphology score	0.70 ± 0.46 1 (0–1)	1.10 ± 0.30 1 (1–2)	1.40 ± 0.49 1 (1–2)	**<0.001** b
Erosion score	0.33 ± 0.47 0 (0–1)	0.67 ± 0.75 0.5 (0–2)	0.87 ± 0.73 1 (0–2)	**<0.001** b
Inflammation	0 (0–0)	0 (0–0)	0 (0–0)	**-**
Necrosis	0 (0–0)	0 (0–0)	0 (0–0)	-

b Overall p-values obtained from Friedman’s repeated-measures analysis of variance by ranks.

Bold values indicate statistical significance (p < 0.05).

**Table 3:** Comparison of septal cartilage specimens stored in ciprofloxacin at days 0, 15, and 30 according to histopathological parameters.

	Day 0	Day 15	Day 30	
Ciprofloxacin	Mean ± SD Median (min–max)	Mean ± SD Median (min–max)	Mean ± SD Median (min–max)	p
Chondrocyte count/cellularity	75.17 ± 25.62 80 (12–116)	56.43 ± 22.73 56 (7–99)	44.90 ± 18.88 42 (4–74)	**<0.001** b
Empty lacunae %	32.83 ± 18.61 27 (9–93)	44.53 ± 18.73 41 (15–96)	58.48 ± 18.71 57 (29–97)	**<0.001** b
Chondrocyte morphology score	0.87 ± 0.73 1 (0–3)	1.27 ± 0.64 1 (0–3)	1.73 ± 0.78 2 (1–3)	**<0.001** b
Erosion score	0.40 ± 0.49 0 (0–1)	0.60 ± 0.72 0.5 (0–3)	0.97 ± 0.80 1 (0–3)	**<0.001** b
Inflammation	0.07 ± 0.36 0 (0–2)	0.10 ± 0.40 0 (0–2)	0.10 ± 0.40 0 (0–2)	0.368 b
Necrosis	0.07 ± 0.36 0 (0–2)	0.10 ± 0.40 0 (0–2)	0.10 ± 0.40 0 (0–2)	0.368 b

b Overall p-values obtained from Friedman’s repeated-measures analysis of variance by ranks.

Bold values indicate statistical significance (p < 0.05).

**Table 4:** Between-solution comparison of changes from baseline (Δ) in histopathological parameters.

		Ethanol	Povidone-iodine	Ciprofloxacin		
		Mean ± SD Median (min–max)	Mean ± SD Median (min–max)	Mean ± SD Median (min–max)	Overall p value	Adjusted post hoc comparison
Chondrocyte count/cellularity	Δ1	−16.13 ± 23.41 −15 (−67) – (41)	−13.66 ± 17.32 −10 (−50) – (22)	−18.73 ± 14.91 −15 (−74) – (6)	0.332 b	—
	Δ2	−28.90 ± 22.53 −28 (−75) – (34)	−21.06 ± 15.91 −19 (−52) – (13)	−30.26 ± 17.73 −34 (−77) – (3)	**0.048** b	Povidone-iodine vs ciprofloxacin (adjusted p = 0.043)
	Δ3	−10.76 ± 16.31 −10 (−53) – (24)	−7.40 ± 10.63 −7 (−24) – (39)	−11.53 ± 10.98 −11 (−35) – (15)	0.465 b	—
Empty lacunae (%)	Δ1	7.73 ± 16.44 5 (−32) – (41)	8.36 ± 9.31 7 (−15) – (27)	11.70 ± 10.64 10 (−9) – (41)	0.185 b	—
	Δ2	21.30 ± 17.54 27 (−19) – (54)	17.18 ± 11.39 20 (−8) – (41)	25.56 ± 16.48 23 (1–61)	**0.032** b	P-I vs CIP (adjusted p=0.029)
	Δ3	13.56 ± 17.36 13 (−24) – (42)	9.43 ± 8.86 8 (−23) – (26)	13.86 ± 13.27 10 (−8) – (62)	0.281 b	—
Chondrocyte morphology score	Δ1	0.36 ± 0.61 0 (0–2)	0.40 ± 0.49 0 (0–1)	0.40 ± 0.49 0 (0–1)	0.570 b	—
	Δ2	1.10 ± 0.54 1 (0–2)	0.70 ± 0.53 1 (0–2)	0.86 ± 0.77 1 (0–2)	0.066 b	—
	Δ3	0.73 ± 0.63 1 (0–2)	0.30 ± 0.46 0 (0–1)	0.46 ± 0.50 0 (0–1)	**0.035** b	No significant pairwise difference
Erosion score	Δ1	0.30 ± 0.53 0 (0–2)	0.33 ± 0.47 0 (0–1)	0.20 ± 0.48 0 (0–2)	0.327 b	—
	Δ2	0.43 ± 0.50 0 (0–1)	0.53 ± 0.50 1 (0–1)	0.56 ± 0.62 0.5 (0–2)	0.681 b	—
	Δ3	0.13 ± 0.43 0 (−1) – (1)	0.20 ± 0.40 0 (0–1)	0.36 ± 0.49 0 (0–1)	0.115 b	—

b Overall p-values obtained from Friedman’s repeated-measures analysis of variance by ranks.

Bold values indicate statistical significance (p < 0.05).

Adjusted pairwise comparisons are shown only when the overall comparison was statistically significant.

Δ1: Day 15 – Day 0; Δ2: Day 30 – Day 0; Δ3: Day 30 – Day 15.
